# Crypt cells are involved in kin recognition in larval zebrafish

**DOI:** 10.1038/srep24590

**Published:** 2016-04-18

**Authors:** Daniela Biechl, Kristin Tietje, Gabriele Gerlach, Mario F. Wullimann

**Affiliations:** 1 Graduate School of Systemic Neurosciences & Department Biology II, Ludwig-Maximilians-Universität Munich, Grosshadernerstr. 2, 82152 Planegg-Martinsried Germany; 2 Department of Biology and Environmental Sciences, Carl von Ossietzky University Oldenburg, Carl von Ossietzky Str. 9-11, 26111 Oldenburg Germany

## Abstract

Zebrafish larvae imprint on visual and olfactory kin cues at day 5 and 6 postfertilization, respectively, resulting in kin recognition later in life. Exposure to non-kin cues prevents imprinting and kin recognition. Imprinting depends on MHC class II related signals and only larvae sharing MHC class II alleles can imprint on each other. Here, we analyzed which type of olfactory sensory neuron (OSN) detects kin odor. The single teleost olfactory epithelium harbors ciliated OSNs carrying OR and TAAR gene family receptors (mammals: main olfactory epithelium) and microvillous OSNs with V1R and V2R gene family receptors (mammals: vomeronasal organ). Additionally, teleosts exhibit crypt cells which possess microvilli and cilia. We used the activity marker pERK (phosphorylated extracellular signal regulated kinase) after stimulating 9 day old zebrafish larvae with either non-kin conspecific or food odor. While food odor activated both ciliated and microvillous OSNs, only the latter were activated by conspecific odor, crypt cells showed no activation to both stimuli. Then, we tested imprinted and non-imprinted larvae (full siblings) for kin odor detection. We provide the first direct evidence that crypt cells, and likely a subpopulation of microvillous OSNs, but not ciliated OSNs, play a role in detecting a kin odor related signal.

Olfaction is an important sense for detection and discrimination of the environment in all vertebrates, including teleosts, such as the zebrafish, *Danio rerio*. In addition to information on the location and composition of food, this sense mediates the recognition of objects of an aversive nature, such as predators, and, most importantly, of social cues such as pheromones^1^.

Olfactory imprinting is a specific learning process during early development that occurs in a short period of time. The life-long memory of the learned cue influences environmental (see for review)^2^, social^3^, dietary^4^ and mating^5^ preferences in a wide variety of species. These memories are critically important, for example young salmon imprint on their natal stream odors and use these memories for spawning migration^6^. Furthermore, some coral reef fish larvae memorize olfactory cues of their natal environment which allows them to return and settle at their home reefs^7^.

Imprinting on olfactory cues plays an important role in the discrimination between ‘own’ and ‘foreign’ in terms of social behavior such as altruism and inbreeding avoidance. The ability to distinguish between kin and non-kin is defined as kin recognition and has been shown in many species (for review see^8^,^9^) including in amphibians^10^,reptiles^11^, birds^12^, mammals^13^ and fish^2^.

Previous studies have shown that zebrafish larvae imprint on visual and olfactory cues of their immediate kin (siblings) during a 24 h time window at 6 days postfertilization (dpf)^14^,^15^,^16^. Larvae can use the learned cues to differentiate between kin and non-kin later in life (kin recognition)^16^. However, imprinting and, consequently, kin recognition does not occur when larvae experience cues of non-kin during the imprinting phase, suggesting a genetic predisposition for kin odor^17^. Further investigations revealed major histocompatibility complex (MHC) peptide ligands to be the underlying chemical cues triggering olfactory imprinting^18^. *In vivo* calcium imaging showed responses to MHC peptides in olfactory bulb neurons to be spatially overlapping with responses to kin odor but not food odors, suggesting MHC peptides to be part of kin odor^18^.

While imprinting is a critical process for salmon (see above), it is still not fully understood when in development imprinting occurs, which cues trigger imprinting or what the underlying genetic basis of imprinting is (reviewed in)^19^. Furthermore, captive rearing changes brain development in salmonids^20^ which might negatively affect the imprinting process^19^. In contrast, the timing of imprinting, the required cues and the genetic basis are already known for zebrafish. In addition, kin recognition, as a result of olfactory imprinting, can be easily detected in laboratory reared animals at 10 days post-hatching. These traits combined make zebrafish an ideal model for studying the mechanisms of imprinting and kin recognition.

The teleost olfactory system lacks a separate vomeronasal organ (VNO) in addition to a main olfactory epithelium. Instead, teleosts possess a single olfactory epithelium (OE) embedded in the nostrils dorsally on each side of the head. Odorants are detected by thousands of different types of olfactory sensory neurons (OSNs) which mediate odor information via the olfactory nerve into the olfactory bulb, the first central nervous station for odor processing.

The two main types of vertebrate OSNs are ciliated (cOSNs) and microvillous olfactory sensory neurons (mOSNs) which in teleosts and mammals express olfactory receptors of the OR and TAAR gene families or V1R- and V2R-type genes, respectively. In addition, teleosts feature two more minor groups of OSN types. Crypt cells, which apparently express only a single olfactory receptor, the V1R-related ORA4^21^ and the recently identified *kappe* neurons^22^, both believed to be absent in tetrapods^23^,^24^.

All four OSN types are recognizable by morphological characteristics like cell- shape, nuclear position within the olfactory epithelium and sometimes by their cell extensions. The cOSN somata are located most basally and extend a long slender dendrite towards the olfactory pit lumen. Cell bodies of mOSNs appear plumper with short dendrites and their nuclei are located at intermediate depths of the OE. Dendrites of cOSNs and mOSNs end in a so-called olfactory knob from which either cilia or microvilli protrude into the olfactory lumen. Compared to cOSNs and mOSNs, crypt cells and *kappe* neurons represent only a small population amongst OSNs but are morphologically well definable as being different from the two main OSN types. Both crypt and *kappe* neurons are apically positioned within the OE directly facing the lumen of the olfactory organ. Crypt cells are ovoid-shaped with a large apical positioned soma and a typical crypt on their apical pole bearing microvilli and cilia^25^. The *kappe* OSN type recently described by the Korsching lab^22^ are somewhat similar to crypt cells but appear more pear-shaped and are positioned even more apical than crypt cells. Moreover, *kappe* neurons do not possess cilia, but only microvilli that protrude on their apical end which is formed like a cap^22^.

Additionally to these morphological characteristics, the use of immunohistochemical markers, such as calcium-binding-proteins, which are often expressed in a cell-type selective manner, facilitates the identification of OSNs. In the zebrafish olfactory system, various calcium-binding-proteins show expression in OSNs as well as in their axonal projections into the olfactory bulb. Moreover, a combinatorial immunohistological expression analysis of four calcium-binding-proteins, that is calbindin, calretinin, parvalbumin and S100, reveals at least eight subpopulations of zebrafish OSNs^26^. As shown before, the calcium binding protein S100 is a marker for zebrafish crypt neurons and a small subpopulation of mOSNs. Although this immunopositivity results mostly from a cross-reaction with an unknown protein^21^,^27^, the S100-like antibody can be used to detect selectively zebrafish crypt cells and a small subpopulation of mOSNs^26^. In addition, the projections of S100 positive OSNs into the olfactory bulb are visualized and are restricted to one single mediodorsal glomerulus (mdG2)^26^,^27^,^28^. Since crypt cells are only present in teleostean and cartilaginous fishes and not in land vertebrates, these cells may have a special role in odor detection and olfactory processing in fish. Because teleosts lack a separate vomeronasal organ, this special olfactory cell type might be involved in recognition of social odorants and resulting behavior.

However, presently the type(s) of OSN(s) detecting kin specific odor in zebrafish are unknown. Therefore, we stimulated the OE of imprinted and non-imprinted larval zebrafish with various odors (food, conspecific odor, kin odor) in a series of experiments. This resulted in differential activation of OSNs which is shown by an increase in the activity marker pERK (phosphorylated extracellular signal regulated kinase) after exposure to the stimuli mentioned. Thus, knowing already the time window of imprinting, some of the likely involved signals and the genetic basis for imprinting, we investigated in the present study which type of olfactory sensory neuron (OSN) detects kin odor.

## Results

### pERK is a reliable marker for showing differentially activated zebrafish olfactory sensory neurons in response to different stimuli

The phosphorylated extracellular signal regulated kinase pERK is used for marking neuronal activity in mammalian olfactory systems^29^. Presence of pERK indicates neuronal activation of the extracellular signal regulated kinase (ERK)/mitogen activated protein kinase (MAPK) signaling pathway caused by a binding of signaling molecules or a synaptic transmitter release^30^,^31^.

**Figure 1:**
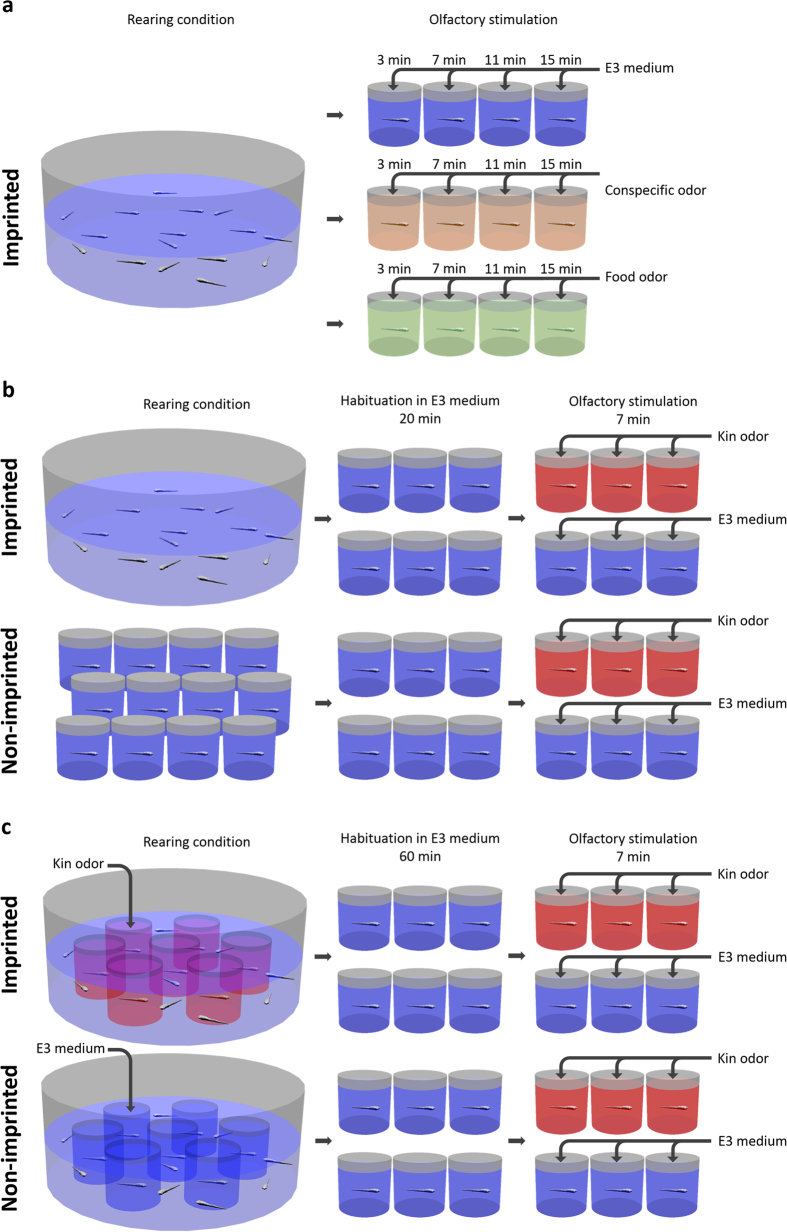
Schemes show three setups of performed experiments. (**a**) Validation of pERK as a marker for olfactory sensory neuron activity. (**b**) Kin odor test I. (**c**) Kin odor test II.

To validate pERK as a neuronal activity marker in the larval zebrafish olfactory epithelium, we performed a temporal analysis of pERK upregulation with two different olfactory stimuli in comparison to control stimulation with E3 medium. This experiment should give information on best duration of stimulation and additionally on the question whether pERK immunofluorescence shows different activation patterns due to different stimuli. We stimulated 9 day old larvae (group reared; see Methods and Fig. 1a) with either non-kin conspecific larvae odor, food odor or, for controls, with E3 medium for 3, 7, 11 and 15 minutes. Afterwards, we used an antibody against pERK to mark activated OSNs within the olfactory epithelium and counted those OSNs using the accepted morphological criteria for the different types of OSNs (see Methods).

**Figure 2:**
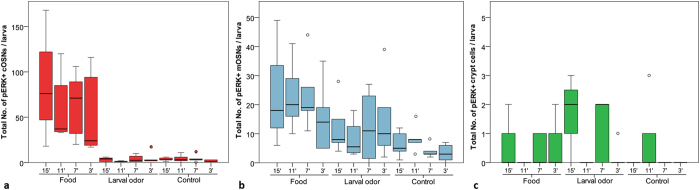
Effect of exposure duration of different stimuli on activity of cOSNs, mOSNs and crypt cells. 9 day old zebrafish larvae were exposed to either food odor, non-kin larvae odor or E3 medium as control (ctr) for either 3, 7, 11, or 15 minutes (min). The total number of pERK+activated cOSNs, mOSNs, and crypt cells was counted per larva and statistically analyzed. Box plots show median, upper and lower quartile and whiskers (maximum interquartile range: 1.5). (**a**) Stimulus duration does not affect number of pERK + cOSNs in larvae stimulated with food odor (Kruskall-Wallis test: H(2) = 0.794, p = 0.851, n_3,7,11 min_ = 5, n_15 min_ = 3), larvae odor (H(2) = 3.030, p = 0.387, n_3,15 min_ = 5, n_7,11 min_ = 4), or in controls (H(2) = 2.866, p = 0.413, n_3,7,11,15 min_ = 5). (**b**) Number of pERK + mOSNs does not alter at different stimulus durations when stimulated with food odor (H(2) = 1.714, p = 0.634), larvae odor (H(2) = 0.964, p = 0.810), or in controls (H(2) = 4.779, p = 0.189). For n values: see (**a**). (**c**) No significant difference in number of pERK+ crypt cells at different stimulus durations when stimulated with food odor (H(2) = 2.488, p = 0.478), larvae odor (H(2) = 6.685, p = 0.083), or in controls (H(2) = 6.316, p = 0.097). For n values: see (**a**).

Intensity of pERK labeled OSNs does not seem to depend on stimulus duration. Equally strongly pERK upregulated OSNs were observed with all four stimulus durations using two odor stimuli and control stimulation (Fig. 2a–c). However, we observed the best signal to noise ratio at stimulus durations of 7 minutes (data not shown). In addition, the duration of stimulation did not show an effect on the number of activated OSN types, because within each OSN type, no significant differences were observed between the four stimulation durations for all three stimuli (Fig. 2a–c).

**Figure 3:**
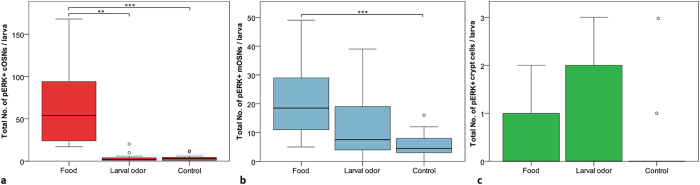
Differential activation of cOSNs, mOSNs and crypt cells by stimulation with different odors. 9 day old zebrafish larvae were exposed to either food odor, non-kin larvae odor or E3 Medium (control) (pooled data of Fig. 2). The total number of pERK+activated cOSNs, mOSNs, and crypt cells was counted per larva and statistically analyzed. Box plots show median, upper and lower quartile and whiskers (maximum interquartile range: 1.5). *indicates statistical significance p: ***p < 0.001. (**a**) cOSNs are strongly activated by food odor. Significantly more pERK+cOSNs were counted in larvae stimulated with food compared to larvae odor (Mann-Whitney U: 4.0, p < 0.001, median (Mdn)_food_ = 54, Mdn_larvae_ = 2, n = 18) and to control (U < 0.0, p < 0.001, Mdn_food_ = 54, Mdn_ctr_ = 3, n_food_ = 18, n_ctr_ = 20). (**b**) mOSNs show the highest activation when stimulated with food odor. Significantly more mOSNS were activated by food odor compared to control stimulation (U: 33.5, p < 0.001, Mdn_food_ = 19, Mdn_ctr_ = 4.5, n_food_ = 18, n_ctr_ = 20). pERK + mOSNs stimulated with larvae odor do not differ in numbers compared to controls (U: 116.5, p = 0.062, Mdn_larvae_ = 7.5, Mdn_ctr_ = 4.5, n_larvae_ = 18, n_ctr_ = 20). (**c**) Crypt cells show no significant difference in pERK+cell numbers due to stimulation with different odors (Kruskall Wallis test: H(2) = 3.197, p = 0.202, n_food_ = n_larvae_ odor = 18, n_ctr_ = 20).

Because we observed that stimulus duration did not affect the number of activated OSNs, we plotted pERK activated cells independent of stimulus durations against the two different odors food and non-kin conspecific larvae odor and compared it with controls (Fig. 3). The activation profile of the different OSNs revealed a highly significant activation in response to food odor compared to controls in ciliated and microvillous OSNs (cOSNs, mOSNs) (Fig. 3a,b). Stimulation with non-kin conspecific larvae odor did not show a significant difference in number of activated neurons compared to control stimulation in both mOSNs and cOSNs. In contrast, crypt cells did not show any significant activation in response to both stimuli compared to controls (Fig. 3c). While mOSNs and cOSNs did show a significant activation in response to food but not to non-kin conspecific larvae odor, crypt cells did not respond to either of the stimuli. These results clearly show (a) that within the temporal range tested, stimulus duration has no effect and (b) that pERK is a reliable marker for activated OSNs in zebrafish larvae specific for different odor stimulations.

Finally, a comparison of activated OSN types depending on different odor stimulation shows the following (Supplementary Fig. 1). After food stimulation, significantly more activated cOSNs and mOSNs are seen compared to crypt cells. There are also higher numbers of activated cOSNs versus mOSNs. After non-kin conspecific larvae odor stimulation, significantly more activated mOSNs and cOSNs are seen compared to crypt cells. Also, a significant higher number of mOSNs were activated in comparison to cOSNs. Somewhat surprisingly (but see discussion), within control stimulations, activated cOSN and mOSN numbers are significantly higher than crypt cell numbers.

### Exposure to kin odor indicates a role of crypt cells and possibly of mOSNs in olfactory kin recognition (and maybe in imprinting) in zebrafish

The results of the following two test series show a role of mOSNs and crypt cells in detecting kin specific odor. Because of the high number of activated crypt cells in imprinted larvae of the *control group* in the first kin odor test (kin odor test I), it can be speculated whether this high activation is due to a higher spontaneous firing rate or, alternatively, to the rest activity of these OSNs resulting from previous kin exposure resulting from the group rearing condition prior to the relatively short adaptation phase in the glass beaker containing E3 medium (Fig. 1b). To answer this question we decided to repeat the experiment (i.e. kin odor test II) with identical rearing conditions of imprinted and non-imprinted groups and with extension of the adaptation period to 1 h before starting the odor stimulation to allow the pERK signal to return to baseline (Fig. 1c). By rearing both groups under the same conditions except for the presence or absence of the olfactory kin-related signals, we also ascertain that differing physiological stress factors did not influence our results. As expected, in this second kin odor test, the high activation of the imprinted control group was eliminated (see discussion).

#### Kin odor test I

**Figure 4:**
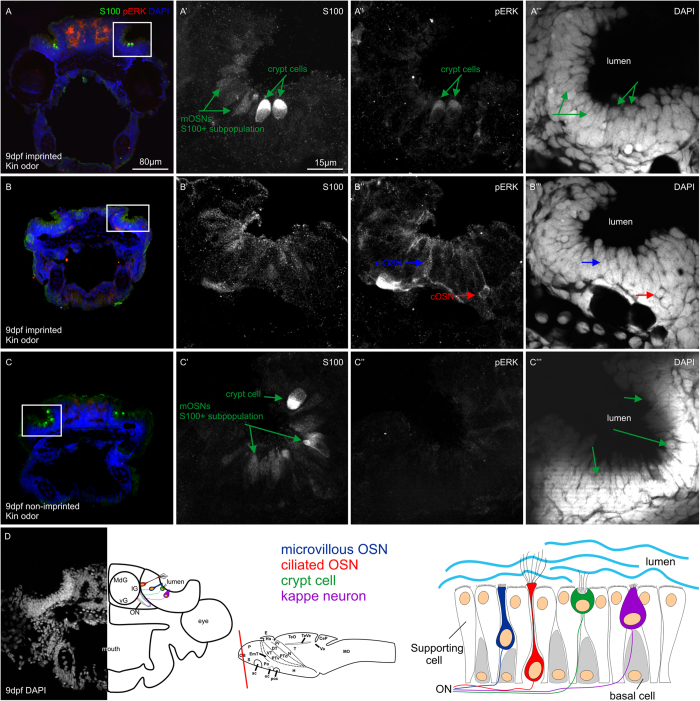
Examples of activated OSN identification and counting. All photographs shown are confocal optical sections. (**A**–**C**) Overviews of 9 dpf larval zebrafish cross-sections triple-stained for DAPI, S100 and pERK. (A’-A”’), (B’-B”’), and (C-C”’) show magnified monochromatic pictures of each marker in the olfactory epithelium. Note examples of activated crypt cells in imprinted larvae tested with kin odor (A-A”’) as well as some mOSNs and cOSNs (B-B”’). In non-imprinted larvae tested with kin odor, crypt cells are not activated (C-C”’). (**D**) shows a DAPI view of the position of the olfactory epithelium relative to eye and olfactory bulb with a corresponding explanatory drawing. Larval brain outline indicates the level of section of (**D**). Drawing at right bottom gives an overview on the cytoarchitectonic organization of the olfactory epithelium. Abbreviations: ac anterior commissure, CeP cerebellar plate, DT dorsal thalamus, E epiphysis, EmT eminentia thalami, H hypothalamus, Ha habenula, lG lateral glomeruli, MdG mediodorsal glomeruli, MO medulla oblongata, N region of the nucleus of the medial longitudinal fascicle, OB olfactory bulb, oc optic chiasma, ON olfactory nerve, P pallium, Po preoptic region, poc postoptic commissure, PTd dorsal part of posterior tuberculum, PTv ventral part of posterior tuberculum, S subpallium, T tegmentum, TeO tectum opticum TeVe tectal ventricle, Va valvula cerebelli, vg ventral glomeruli, VT ventral thalamus.

In this experiment we investigated which OSN type(s) respond to a kin odor produced by full siblings and therefore play a role in olfactory kin recognition and maybe imprinting. To this aim, we stimulated 9 dpf old imprinted (group reared, see Methods) and non-imprinted (isolated reared; see Methods and Fig. 1b) larvae with kin odor for 7 minutes, and compared these two groups with equally reared control groups stimulated with E3 medium. In this experiment, we used the anti-pERK antibody together with an established crypt cell marker, an antibody against the calcium binding protein S100^26^, in order to examine a possible overlap between these two markers (Fig. 4) and to differentiate S100-positive (S100 +) from S100-negative pERK activated OSNs.

**Figure 5:**
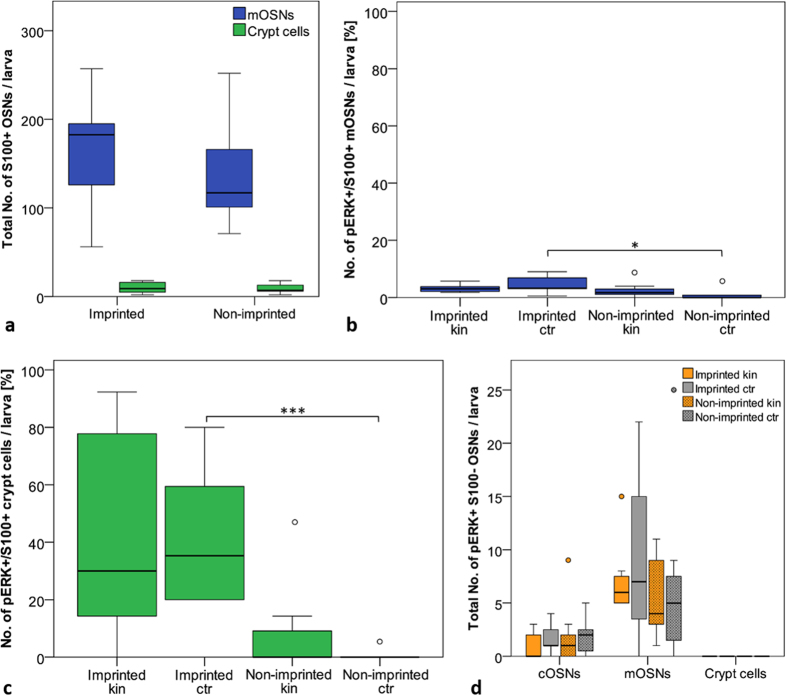
Kin odor test I (see Fig. 1b): Effects of olfactory imprinting. (**a**) Total cell quantity of S100+mOSNs and crypt cells. Box plots show median, upper and lower quartile and whiskers (maximum interquartile range: 1.5). No significant difference in total number of mOSNs and crypt cells was found (mOSNs Mann-Whitney U: 104.5, p = 0.109, Mdn_impr_ = 182.5, Mdn_non impr_ = 117; crypt cells U: 149, p = 0.894, Mdn_impr_ = 9, Mdn_non impr_ = 7, n_impr_ = 18, n_non impr_ = 17). **(b)** S100+/pERK+mOSNs shown as percentage of all S100 + mOSNs per larva. Box plots show median, upper and lower quartile and whiskers (maximum interquartile range: 1.5). *indicates statistical significance p: *p < 0.05, *p < 0.01, ***p < 0.001 (also applies to (**c**) S100+mOSNs show no difference in activation between imprinted and non-imprinted larvae after kin stimulation. Number of activated mOSNs is significantly higher in imprinted larvae versus non-imprinted control larvae (Mann-Whitney U < 0.001, p < 0.001, Mdn_impr_ = 3.25, Mdn_non impr_ = 0, n_impr_ = 11, n_non impr_ = 7). (**c**) S100+/pERK+crypt cells shown as percentage of all S100 + crypt cells per larva. S100 + crypt cells show no difference in activation between imprinted and non-imprinted larvae after kin stimulation U: 15, p = 0.035 [Bonferroni correction], Mdn_impr_ = 30, Mdn_non impr_ = 0, n_impr_ = 7, n_non impr_ = 10). A significant difference between imprinted and non-imprinted control larvae exists (U < 0.001, p < 0.001, Mdn_impr_ = 35, Mdn_non impr_ = 0, n_impr_ = 11, n_non impr_ = 7). (**d**) The total numbers of pERK activated, but S100 negative cOSNs, mOSNs, and crypt cells are shown. Box plots show median, upper and lower quartile and whiskers (maximum interquartile range: 1.5). *indicates statistical significance p: **p < 0.01. No difference in cell activation was found in either cOSNs (Kruskall-Wallis H(2) = 1.729, p = 0.630) or mOSNs (H(2) = 1.901, p = 0.593) (n_impri kin_ = 7, n_impr ctr_ = 11, n_non impr kin_ = 10, n_non impr ctr_  = 7).

First, we were interested if there was a quantitative difference of S100 + mOSNs and crypt cells between imprinted and non-imprinted larvae (Fig. 5a). When plotting all detected S100 + mOSNs and crypt cells independent of pERK immunopositivity, no significant difference between imprinted and non-imprinted larvae was observed. Imprinted as well as non-imprinted larvae show nearly the same amount of S100 + mOSNs or crypt cells. Therefore, we can exclude that preventing olfactory imprinting in zebrafish larvae has an effect on total OSN cell number (i.e. S100 + mOSN and crypt cell) development within the olfactory epithelium.

Focusing on the two populations of S100 + OSNs, we plotted the percentage (Fig. 5b,c) of activated (pERK +) S100 positive mOSNs and crypt cells per larva. Regarding S100+ mOSNs as well as crypt cells, all S100 + OSNs of each larva were counted and therefore the percentage of activated S100 + OSNs can be specified. The number of double labelled mOSNs was significantly higher in imprinted larvae than in non-imprinted larvae after exposure to E3 medium (see Fig. 5b). This might result from group rearing conditions and time of adaptation before stimulation (see Discussion).

The pERK+/S100+double labelled crypt cells showed a clearer picture of differential activation after kin odor versus E3 medium exposure (Fig. 5c). Stimulation with kin odor showed considerable numbers of activated crypt cells in imprinted larvae whereas in non-imprinted larvae only few activated crypt cells were counted. However, again, as for double labelled mONSs, also imprinted control larvae stimulated with E3 medium showed considerable numbers of activated crypt cells and the difference is highly significant compared to non-imprinted control larvae (Fig. 5c), likely for the same reasons as indicated for double labelled mOSNs (group rearing and adaptation time before testing; see Discussion).

Finally, we counted all other pERK+cell types within the olfactory epithelium which were S100 negative using the accepted cytological and morphological criteria for OSNs (see Methods). Exposure to either kin odor or E3 medium showed no difference in pERK activation of ciliated S100 negative OSNs in imprinted and non-imprinted larvae (Fig. 5d). In S100 negative mOSNs of imprinted and non-imprinted larvae olfactory stimulation with kin odor or E3 medium showed no significant effect on activation of OSNs. As expected, no S100 negative crypt cells were detected which were pERK immunoreactive. In summary, there are no significant differences in S100 negative OSNs between kin odor stimulated imprinted and non-imprinted larvae in this experiment.

#### Kin odor test II

**Figure 6:**
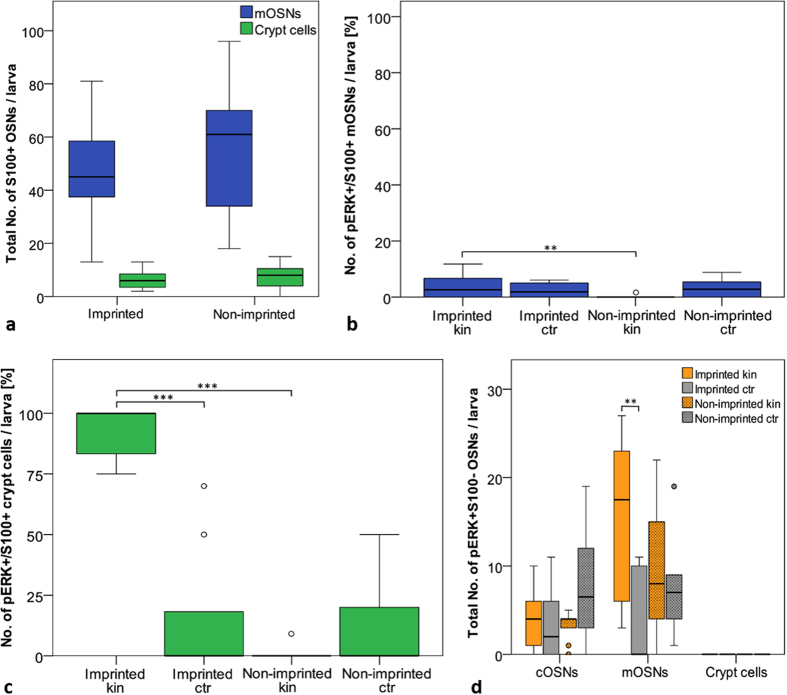
Kin odor test II (see Fig. 1c): Effects of olfactory imprinting. (**a**) Total cell quantity of S100 + mOSNs and crypt cells. Box plots show median, upper and lower quartile and whiskers (maximum interquartile range: 1.5). Imprinting has no effect on total cell numbers (mOSNs U: 147, p = 0.227, Mdn_impr_ = 54, Mdn_non impr_ = 61; crypt cells U: 156, p = 0.351, Mdn_impr_ = 6, Mdn_non impr_ = 8, n_impr_ = 19, n_non impr_ = 20). (**b**) S100 +/pERK + mOSNs shown as percentage of all S100 + mOSNs per larva. Box plots show median, upper and lower quartile and whiskers (maximum interquartile range: 1.5). *indicates statistical significance p: *p < 0.05, *p < 0.01, ***p < 0.001 (also applies to (**c**)). Significantly more S100 + mOSN are activated in imprinted larvae versus non-imprinted control larvae exposed to kin odor (U: 18, p = 0.008, Mdn_impr_ = 2.6, Mdn_non impr_ = 0, n_impr_ = n_non impr_ = 10). (**c**): A significant higher number of crypt cells are activated after kin odor stimulation in imprinted compared to non-imprinted larvae (U < 0.001, p < 0.001, Mdn_impr_ = 100, Mdn_non impr_ = 0, n_impr kin_ = n_non impr kin_ = 9) and compared to imprinted control larvae stimulation (U: U < 0.001, p < 0.001, Mdn_impr_ = 100, Mdn_non impr_ = 0, n_impr kin_ = 10, n_impr ctr_ = 9). No difference in activation was found within non-imprinted larvae. (**d**) The total numbers of pERK activated, but S100 negative cOSNs, mOSNs, and crypt cells are shown. Box plots show median, upper and lower quartile and whiskers (maximum interquartile range: 1.5). * indicates statistical significance p: **p < 0.01. Cell activation was similar for all treatments in cOSNs (H(2) = 5.405, p = 0.144) (n_impri kin_ = 10, n_impr ctr_ = 9, n_non impr kin_ = 10, n_non impr ctr_ = 10). A significantly higher number of mOSNs was found in imprinted larvae stimulated with kin compared to control stimulation (Mann-Whitney U: 13, p = 0.008, Mdn _impr kin_ = 17.5, Mdn_impr ctr_ = 0). No S100- negative crypt cells were observed.

As in the first experiment using kin odor stimulation, there is no significant difference in total quantity of S100+mOSNs and crypt cells between imprinted and non-imprinted larvae (Fig. 6a).

The small S100+subpopulation of mOSNs and crypt cells which are also positive for the activity marker pERK are shown as percentage of all S100+OSNs counted (Fig. 6b,c). In the case of S100+/pERK + mOSNs, the generally very low cell numbers reveal a significant difference between imprinted and non-imprinted larvae when exposed to kin odor (Fig. 6c). In contrast to S100+/pERK + mOSNs, crypt cells of imprinted larvae show high numbers of pERK activated cells in response to the kin odor (Fig. 6c). In imprinted larvae exposed to kin odor, more than 90% of all S100+crypt cells were activated, which is highly significantly more than in non-imprinted larvae exposed to kin odor and than in imprinted larvae exposed to E3 medium. Little activation was also observed in non-imprinted larvae exposed to E3 medium. In addition, extending the adaptation time in E3 medium before starting the stimulation experiments in all groups reduced greatly the high amount of activated crypt cells (and presumably also mOSNs) in imprinted control larvae as seen in the experiment before (see Fig. 5c). Therefore we can exclude a higher spontaneous activity of crypt cells in imprinted versus non-imprinted larvae.

Comparing data on S100 negative OSNs that were activated in response to kin water or E3 medium in this and the previous experiment revealed a similar picture (Figs 5d and 6d). As expected, no S100 negative/pERK positive crypt cells were observed, confirming that all of them are S100 positive. Ciliated S100 negative OSNs of imprinted larvae showed only slight activation in response to the kin odor. However, a certain number of cOSNs of non-imprinted larvae were also activated in response to the kin odor. Also imprinted and non-imprinted control larvae show small numbers of activated cOSNs. However, there were no significant differences between cOSNs among all four tested groups (Fig. 6d). Regarding mOSNs, higher numbers of S100 negative mOSNs are pERK activated in imprinted as well as non-imprinted larvae in response to kin odor stimulation or E3 medium stimulation. In this second experiment, there is a significant difference in numbers between the imprinted kin and imprinted control group (see Discussion).

**Figure 7:**
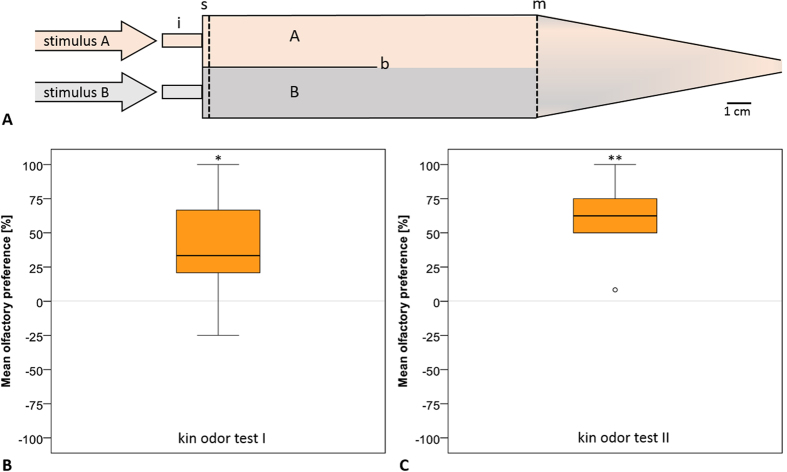
Group reared full-sibling larvae prefer kin odor over non-kin odor in behavior test. Mean olfactory preference of larvae was tested using the two-channel choice flume. (**A**) Two-channel choice flume (after Hinz *et al.*)^18^. Two distinct parallel-flowing water masses A (orange) and B (grey) containing different odors are separated through a glass barrier (b). A sponge (s) reduces pulsation caused by the pump. i: inflow; m: mesh screen to prevent test fish leaving the area of laminar flow. (**B**) Full-siblings of test larvae (11 dpf) of kin odor test I (compare Fig. 1b) significantly prefer the smell of kin over non-kin (Wilcoxon signed-rank test Z: -2.325, p = 0.020, median (Mdn) = 33.3, n = 11). (**C**) Full-siblings of test larvae (9dpf) of kin odor test II (compare Fig. 1c) showed a significant olfactory preference for kin compared to non-kin odor (Wilcoxon signed-rank test Z: -3.065, p = 0.002, Mdn = 62.5, n = 12).

Finally, in both kin odor experiments, we tested 11 dpf or 9 dpf old larvae in a 2 channel choice flume (as established in the Gerlach laboratory^15^,^16^,^32^; Fig. 7) which showed successful imprinting in both of these group reared larvae taken from the same batch, respectively, as the larvae used for the stimulation experiment.

## Discussion

We investigated in zebrafish larvae whether pERK expression in OSNs depends on/differs with olfactory stimulus duration and whether different olfactory stimuli result in a differential activation pattern of pERK. Therefore, in a first experiment, we stimulated group raised zebrafish larvae at 9 dpf (see Methods and Fig. 1a) to validate pERK as a marker of OSN activity in response to a food odor or a non-kin conspecific larvae odor at various exposure times. Our results demonstrate that pERK is a reliable marker to show differentially activated OSN types after exposure to different odors (Figs 2 and 3). The pERK signal was rapidly induced and detectable in different types of OSNs after 3 minutes of odor stimulation (Fig. 2). The intensity of immunofluorescence as well as the numbers of OSNs remained unchanged with prolonged odor exposure times (7, 11, 15 min). Both cOSNs and mOSNs were strongly activated by the food stimulus compared to controls, while crypt cells were not (Figs 2 and 3). The comparison of OSN types convincingly showed that cOSNs were more strongly activated by food than mOSNs, whereas mOSNs were more strongly activated by (non-kin) conspecific larvae odor compared to cOSNs (Supplementary Fig. 1). These two OSN types occur in equally high numbers already in the larvae, whereas the crypt cells form a minor population (see below). Thus, we interpret the apparent significant differences within the controls simply as a consequence of the much higher numbers of both cOSNs and mOSNs compared to crypt cells.

There is great interspecific variability within teleosts regarding the potential roles of OSNs^33^. In channel catfishes, a comparison of OSN olfactory bulb projections and electrophysiological responses to amino acids and nucleotides (both indicative of food) or bile salts (presumably social signals) in the olfactory bulb indicated that cOSNs respond to amino acids and bile salts, mOSNs to amino acids and nucleotides, and crypt cells to amino acids^34^. Studies in carp indicate that mOSNs are related to feeding, cOSNs to alarm reaction and crypt cells to reproduction^35^. In goldfish, mOSNs expressing V2R-type odorant receptors are best tuned to amino acids^36^. In zebrafish, cOSNs are associated with sensing bile salts and prostaglandins, mOSNs with sensing amino acids and nucleotides, and crypt cells with sensing skin extract (reviewed in^33^,^37^). Koide *et al.*^37^ found in transgenic zebrafish lines visualizing different OSN types that only ablation of mOSNs through genetically encoded tetanus toxin abolished behavioral responses to amino acids. Physiological preference for amino acids by mOSNs was also found in zebrafish^38^. In salmon, mOSNs have also been related to amino acids, while cOSNs sense bile salts^39^,^40^. Trout crypt cells have been related to sensing gonadal extracts^41^. However, in other teleost species, amino acid sensing was clearly also seen in cOSNs^33^,^42^. Thus, a general conclusion that teleost mOSNs mediate food-related olfactory cues based on amino acid detection and cOSNs detect social signals through bile salt sensing is too simplified, because studies in different teleost species show that cOSNS, mOSNs as well as crypt cells respond to amino acids. Our results are consistent with an activation of both cOSNs and mOSNs through food stimulus which contains a variety of chemicals including amino acids. Moreover, non-kin conspecific odor additionally activated mOSNs. In any case, our first experiment ascertains that pERK is a reliable marker for OSN activity in zebrafish larvae after odor stimulation.

In order to investigate which OSNs are involved in kin odor detection, we stimulated in two additional experiments imprinted and non-imprinted larvae (see Methods and Fig. 1b,c) with kin odor containing E3 medium. Here we used an anti-S100 antibody to mark specifically all crypt cells as well as a small subpopulation of mOSNs^26^ in addition to the anti-pERK antibody to stain neuronal activation after odor stimulation. Imprinted zebrafish larvae recognize their kin siblings while non-imprinted larvae do not (15,16; see Introduction). This difference between imprinted and non-imprinted larvae might depend on activity at the level of the olfactory epithelium. Indeed, our results show a great difference between imprinted and non-imprinted larvae with regard to crypt cell activation in response to kin odor in both experiments (Figs 5 and 6). The S100 staining allows for counting all crypt cells and the subpopulation of S100 positive mONS. Since these cell numbers are the same (9 dpf larvae), we can exclude that this highly significant difference is due to a dissimilar number of crypt cells between imprinted and non-imprinted larvae (Fig. 5).

Regarding to the role of crypt cells in olfactory imprinting we compare now in more detail the differences of OSN activation responses of imprinted and non-imprinted larvae after kin odor stimulation between the two experiments using kin odor stimulation. First, high numbers of activated crypt cells were seen in both experiments in kin odor stimulated imprinted fish. However, the first kin odor experiment indicated a possible difference of crypt cells as well as S100 + mOSNs in their spontaneous activity also in control conditions (neutral E3 medium as stimulus) between imprinted and non-imprinted larvae (Fig. 5b,c). To test this possibility we performed a second experiment in which both larvae groups (imprinted and non-imprinted fish) were raised in the same way in glass beakers. Further, the adaptation time was extended (to 1 h) before starting the experiment. Thus, we excluded the possibility that group reared and isolated reared larvae undergo different stress levels (e.g. through water changes using pipettes). Furthermore, the prolonged adaptation time was introduced to make sure that all fish reached baseline levels regarding OSN activity. The data showed clearly that the increased activity seen in crypt cells and mOSNs in imprinted controls in the first experiment (Fig. 5b,c) is eliminated by these changes (Fig. 6b,c). This demonstrates even more explicitly the role of crypt cells in kin recognition.

Besides crypt cells, we marked a significant activation of S100 negative mOSNs (Fig. 6d) in imprinted larvae in response to the kin odor which indicates also an involvement of mOSNs in kin recognition. A collaboration between two OSN types conveying a kin-related signal with subsequent behavioral response is more likely than the involvement of one cell type and is similarly seen in rodents^43^. In contrast, cOSNs did not show significant responses to kin odor (Figs 5d and 6d). Moreover, the kin odor containing E3 medium (see Methods) is doubtlessly comprised of many odor cues, some of which may not be kin-related. Apparently, crypt cells express only a single V1R homologue odorant receptor, encoded by the *ora4* gene^21^. There is strong evidence from other studies in teleosts which implicate a role of crypt cells in reproductive behavior^35^,^44^. In the crucian carp, the number of crypt cells varies during the year, with a dramatic increase during the spawning season^44^. Similar studies in adult zebrafish and guppies did not indicate a seasonal change in crypt cell quantity which might be related to year round reproductive behavior^23^,^45^,^46^. However, the kin-specific ligand(s) and its (their) molecular nature by which crypt (or other) cells are activated is unknown.

Sandulescu and colleagues^47^ report that the zebrafish crypt cell population undergoes nonlinear growth during larval development. This study reports a linear increase of zebrafish crypt cell numbers from day 2 until day 7 of larval postembryonic development, followed by a rapid decrease of crypt cell numbers around 8-9 dpf. Thus, a peak in crypt cell number is reached at 7 dpf with an average of 7.8 cells per larva, with numbers decreasing at 8 dpf and 9 dpf to finally 2.2 cells per larva. At 12 dpf a rebound of crypt cell numbers is seen^47^. Our high crypt cell numbers at 9 dpf (average of 7 cells per larva) may at first glance seem to disagree with these results of a time point of extreme reduction of crypt cells. However, larvae of the other study were maintained at 28 °C while we raised the larvae at 26 °C. Since the development of larvae is temperature dependent^48^, our larvae at 9 dpf are likely delayed in development which might explain our higher cell numbers. We are confident about our numbers of counted crypt cells since they originate from 40 larvae (80 olfactory epithelia) compared to 6 specimens (12 olfactory epithelia) used in the study of Sandulescu and colleagues. Thus, the crypt cell population likely grows linearly until the critical period of imprinting to ensure an adequate amount of cells expressing specific receptors for binding of kin-specific ligands.

Together, these results provide the first direct evidence that clearly crypt cells play a role in detecting a kin odor related signal. They also harbour the possibility that a subpopulation of mOSNs might be involved in kin recognition. The data show that the total numbers of S100 positive mOSNs and crypt cells do not differ between imprinted and non-imprinted fish. Furthermore, there are different quantitative patterns of how cOSNs, mOSNs and crypt cells are activated in response to food and non-kin (conspecific) odor.

## Methods

### Study animals and rearing conditions

Adult zebrafish wildtype were obtained from different commercial breeding facilities (Germany, Vietnam, Sri Lanka) and maintained in 3 liter aquaria per breeding pair at 26 °C under a 13 h:11 h light:dark cycle. Fish were fed daily, alternating with commercial flake food, *Artemia salina* and white mosquito larvae. For breeding spawning trays were used. Eggs were kept in E3 medium^49^ in an incubator at the same temperature and light conditions as the adults. Larvae hatched at 3-4 day post fertilization (dpf). After depletion of the yolk (on 5 dpf) larvae were fed with commercial fry food and *Paramecium spec*. Eggs and larvae were reared according to experiment conditions (see Fig. 1).

Animal Use and Care Protocols were approved by the Institutional Animal Care and Use Committee of the University of Oldenburg and the government of the state Niedersachsen, Germany (18.01.2013-17.01.2016). All experiments were carried out in accordance with the approved guidelines. After the experiment, larvae were killed by an overdose of MS222 (see below).

### Odor choice test

Olfactory preference tests were conducted in a two-channel choice flume (Fig. 7A) with a steady driven flow (30 ml/min per channel; approx. 2.5 cm/sec) generated by a peristaltic pump. Regular dye tests ensured that the flume maintained two distinct parallel-flowing water masses (A and B), which remained entirely separated up to the downstream mesh screen.

For the tests, single fish were placed into the flume with both water sources (kin odor and non-kin odor) running and were allowed to acclimate and swim freely. The test period started directly after the fish experienced both water masses (i.e. entered once both A and B).

We recorded the position of the fish’s head and nose in one or the other water flow every 10 s during two 2-min periods separated by a 1.5-min transition period to switch water sources as a control for possible (non-olfactory) side bias of the fish. If the larvae swam directly at the center line between both water masses, the location would be recorded as ‘unclear’ and excluded from the analysis. The tests were run blind, so that the observer did not know on which side the respective odor stimulus was delivered. Olfactory preference is expressed as the percentage of observations spent in kin odor minus non kin odor stimulus. A random distribution across water masses (zero difference) is expected if a fish did not express a preference for one of the odor stimuli; a negative value indicates a preference for non-kin odor, and a positive value for kin odor (Fig. 7B,C).

Kin odor was created by keeping 25 full siblings of the test fish in 250 ml odorless E3 medium overnight, which then was filled up to 5 l (5 larvae/l). Larvae of genetically different families were used to create the non-kin (conspecific) larvae stimulus.

### Stimulation experiments

#### Validation of pERK as a marker for olfactory sensory neuron activity

Larvae were reared in a group of full siblings (Fig. 1a). At the age of 9 dpf they were olfactory stimulated either with a non-kin conspecific odor mix (generated from three non-related larvae batches of the same age), food odor (generated from commercial flake food and *Paramecium spec*.), or E3 medium. While stimulated for 3, 7, 11, or 15 minutes, single larvae were kept in small glass beakers in a calm environment.

#### Kin odor test I

For the first kin odor stimulation experiment larvae were either reared in olfactory isolation to suppress the imprinting process or in a group of full siblings to evoke imprinting on kin (Fig. 1b). For olfactory isolation single eggs were reared in small glass beakers. At 9 dpf larvae were olfactory stimulated. Thus, single larvae were placed into small glass beakers containing pure E3 medium and were allowed to acclimate for 20 minutes. Afterwards, the olfactory stimulus, either kin odor or E3 medium, was added for 7 minutes. To make sure imprinting was successful, some of the group reared larvae underwent the odor choice test at the age of 11 dpf as described above.

#### Kin odor test II

Larvae were reared in isolation and either visually and olfactory exposed (imprinted) or only visually exposed (non-imprinted) to their kin. Thus, single eggs were placed into small glass beakers. Glass beakers were placed into a larger dish containing 12 eggs from the same batch (Fig. 1c). Larvae that were allowed to imprint on their kin were olfactory stimulated with kin odor at 5 dpf in the evening, at 6 dpf in the morning, noon, and evening and at 7 dpf in the morning. Whereas those larvae, in which imprinting was prevented, were exposed to E3 medium instead of kin odor at corresponding time points. Both groups were able to see their kin during the entire experiment.

Prior to the olfactory stimulation, larvae were placed into fresh glass beakers containing E3 medium and were allowed to acclimate for one hour. Thereafter, they were stimulated for 7 minutes either with kin water or E3 medium. Stimulation took place at 9 dpf. Some of the larvae that were allowed to swim freely in the larger dish, surrounding the small glass beakers, were tested for olfactory preference.

### Tissue preparation and immunohistochemical processing

Larvae were killed with an overdose of tricaine methanesulfonate (MS222; Sigma-Aldrich) in E3 medium and cut in halfes. Heads were fixed with cold 4% PFA overnight and tails transferred into 99% ethanol for later genotyping. Following cryoprotection in 30% sucrose solution overnight, heads were embedded in Tissue-Tek (tissue freezing medium, Leica Jung) and horizontal cryosections of 14 μm thickness were thaw mounted onto Superfrost Plus slide glasses (Thermo Scientific).

Incubations were done in a humid chamber. After washing off TissueTek in PBS, cryosections were incubated in 100% MeOH for 10 minutes at −20 °C, washed several times in PBT and blocked in blocking buffer (2% normal donkey serum, 0.1% fish gelatine, 0.5% Tween 20, 0.5% Triton X-100 in PBS) for 1 h at room temperature. Double labeling with two primary antibodies from same host species, Fab-Fragments (CyTM3-conjugated AffiniPure Fab Fragment, dk-anti-rb IgG (H + L), 1:100 dilution, Jackson Immuno Research) were used.

Slides were incubated with first primary antibody (rabbit anti-pERK, 1:200 dilution, Cell Signaling) diluted in blocking buffer for 2 days at 4 °C followed by incubation with Fab Fragments overnight at 4 °C. Afterwards, slides were incubated with the second primary antibody (rabbit anti-S100, 1:600 dilution, Dako) diluted in blocking buffer for 2 days at 4 °C, following incubation with the second secondary antibody (Alexa 488 anti rabbit, 1:400 dilution, Dianova) diluted in blocking buffer for 2 h at room temperature. Finally, sections were washed in PBT and counterstained with DAPI (40−6-diamidino-2-phenylindole; 1:1000 dilution, Carl Roth) and mounted with Vectashield (Vectorlabs) and coverslipped.

### Confocal microscopy

Optical sections were acquired with a Leica TCS SP-5 confocal laser-scanning microscope (Leica Microsystems). All microscopic images used in this study were processed to RGB stacks and projections by using ImageJ and slightly adapted for brightness and contrast with either ImageJ or Corel PHOTO-PAINT. Photographic plates were mounted and further processed into figures with CorelDRAW 12.0 (Corel Corporation).

### Quantification of activated cells

Stacks of olfactory epithelia were analyzed by using the RoiManager tool of ImageJ. Activated cells (pERK+) were identified according to accepted criteria for OSNs as follows:

Position of cell-soma: basal for ciliated-, intermediate for microvillous- and superficial for crypt- cells. Shape of cell-soma: ciliated OSNs, stout with one long dendrite towards luminal surface; microvillous OSNs, somewhat elongated with basal and superficial dendrite; crypt cells, round with acentric nucleus, no dendrites, but superficial indentation. In addition to the mentioned criteria, the calcium binding protein S100 was used to label crypt cells and a small subpopulation of microvillous cells (as previously described)^26^.

Cell counting for statistical analysis was performed blind, by two observers unknowingly which specimen (imprinted/non-imprinted) and stimulus (control/kin odor) they were evaluating.

### Statistical evaluation

To study the effect of exposure duration on the activity of cOSNs, mOSNs and crypt cells (Fig. 2a–c) the number of pERK+activated cells was counted and a Kruskall-Wallis test was performed (H(2): Chi square value; p: significance value; n: sample size). Differences in pERK activation of cOSNs were first analyzed within the group of food stimulated larvae between different stimulation times. The same test was performed with non kin-larval odor and control stimulation. The activity of mOSNs and crypt cells was analyzed likewise.

Data of different stimuli duration were pooled (Fig. 3) due to the fact that no duration-dependent differences in pERK activation was found. A Kruskall-Wallis test was used to analyze differential activation of cOSNs by stimulation with food, larval odor and control. Followed by a pairwise Mann-Whitney U test including Bonferroni correction for multiple comparison (U: Mann-Whitney U value; Mdn: median). The same procedure was implemented for mOSNs and crypt cells.

In kin odor tests I and II (Figs 5 and 6) the cell quantity of S100-positive (s100+) mOSNs and crypt cells between imprinted and non-imprinted larvae (Figs 5a and 6a) was tested by using a pairwise Mann-Whitney U test. A Kruskall-Wallis test followed by a Mann-Whitney U test including Bonferroni correction was used to analyze the activation of S100+mOSNs (Figs 5b and 6b) and crypt cells (Figs 5c and 6c) between imprinted and non-imprinted larvae either stimulated with kin odor or control. S100-negative OSN numbers were tested likewise (Figs 5d and 6d). Note that only S100 negative mOSNs and cOSNs exist, but no crypt cells.

Olfactory preference is expressed as a preference index (Fig. 7B,C). The percentage of time the larvae spend in kin odor was subtracted by the percentage of time spend in non-kin odor. A Wilcoxon signed-rank test (Z: Wilcoxon signed-rank value) was performed to test whether the preference index differs significantly from zero.

All analyses are two-tailed and were done in IBM SPSS statistic 23 for windows.

## Additional Information

**How to cite this article**: Biechl, D. *et al.* Crypt cells are involved in kin recognition in larval zebrafish. *Sci. Rep.* **6**, 24590; doi: 10.1038/srep24590 (2016).

## Supplementary Material

Supplementary Information
