# Supplementary material for: Crypt cells are involved in kin recognition in larval zebrafish
Source: Sci Rep. 2016 Apr 18;6:24590. doi: 10.1038/srep24590 (PMC4834543; doi:10.1038/srep24590)

Crypt cells are involved in kin recognition in larval zebrafish

Daniela Biechl[1], Kristin Tietje[2], Gabriele Gerlach[2], Mario F. Wullimann[1*]

Supplementary Information: Supplementary Figure 1:

Effects of food odor, non-kin larvae odor and E3 medium stimulation on OSN activation. The total number of pERK+ activated cells (cOSNs, mOSNs, and crypt cells) was counted per larva (pooled data of figure 1) and statistically analyzed. Box plots show median, upper and lower quartile and whiskers (maximum interquartile range: 1.5). * indicates statistical significance p: **p<0.01, ***p<0.001. After food odor stimulation significantly more cOSNs and mOSNs were activated compared to crypt cells (cOSNs - crypt: Mann-Whitney U < 0.001, p < 0.001, Mdn_{cOSNs} =54, Mdn_{crypt}= 0, n = 18; mOSNs – crypt: U < 0.001, p < 0.001, Mdn_{mOSNs} =18.5, Mdn_{crypt}= 0, n = 18). Non-kin larvae odor stimulation activated significantly more mOSNs compared to cOSNs (U: 63.5, p = 0.002, Mdn_{mOSNs} = 7.5, Mdn_{cOSNs} = 2, n = 18) and crypt cells (U: 16, p > 0.001, Mdn_{mOSNs} = 7.5, Mdn_{crypt} = 0, n = 18). Furthermore, a higher number of mOSNs and cOSNS were activated by control stimulation in comparison to crypt cells (mOSNs - crypt: U: 17, p < 0.001, Mdn_{mOSNs} = 4.5, Mdn_{crypt} = 0; cOSNs – crypt: U: 23, p < 0.001, Mdn_{cOSNs} = 3, Mdn_{crypt} = 0, n = 20).

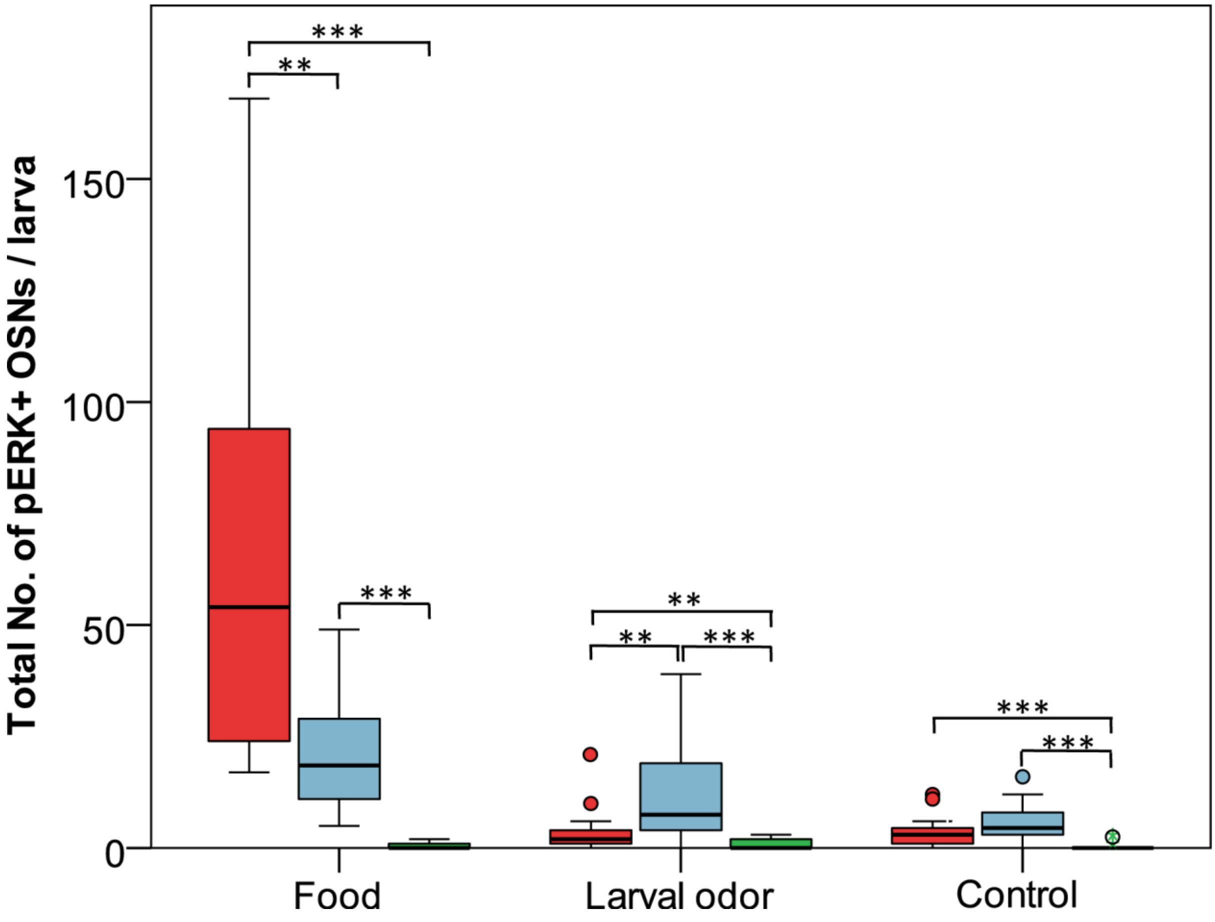

Supplement: Supplementary Information [file srep24590-s1.pdf]
